# Which prenatal biomarker is most appropriate for methylmercury dose-response for neurodevelopmental effects?

**DOI:** 10.1080/10937404.2024.2444650

**Published:** 2024-12-20

**Authors:** Leonid Kopylev, Michael Dzierlenga, Yu-Sheng Lin, Rebecca Nachman, Elizabeth Radke, Hongyu Ru, Deborah Segal

**Affiliations:** Center for Public Health and Environmental Assessment, Office of Research and Development, US Environmental Protection Agency, Washington, USA

**Keywords:** Maternal hair and blood, cord blood, developmental neurotoxicity, methylmercury, biomarkers of MeHg exposure

## Abstract

Developmental neurotoxicity (DNT) is a well-established hazard attributed to methylmercury (MeHg) exposure. This evidence is based primarily upon includes studies that measured biomarkers of MeHg exposure in samples of maternal hair and blood, and cord blood. The aim of this review was to investigate which of these prenatal biomarkers is most appropriate for quantifying the DNT effects attributed to MeHg. A comprehensive literature search covered MeHg dose-response literature published 1998–2022. Studies were evaluated for risk of bias and study sensitivity using IRIS approach. Quantitative results of investigations were extracted and statistically compared. Seven studies were identified that measured both maternal hair and cord blood Hg levels. In these investigations, several DNT umbrella tests and their sub-tests results were modeled. Cord blood MeHg was more sensitive, producing larger estimates of MeHg potency, in most of the comparisons (91%) with maternal hair MeHg estimates for the same sub-tests in the same study. When comparing results from cord blood Hg to maternal hair Hg there was a 75% increase in sensitivity (range: 4–583%). In the two domains where results for maternal hair Hg were more sensitive, the rise was only 18% (Range: 7–29%). There were limited data (two studies) that compared maternal blood and maternal hair biomarkers (maternal blood Hg was more sensitive (mean 320% and range 43–855%) and cord blood biomarkers (maternal blood Hg was more sensitive by approximately 30%). Maternal hair Hg remains an appropriate biomarker for exposure monitoring in many populations, but these data suggest that cord blood Hg is more appropriate for dose-response modeling of MeHg DNT effects.

## Introduction

Long-standing international research demonstrated that exposure to methylmercury (MeHg) (an organic form of the heavy metal mercury (Hg)) is associated with developmental neurotoxicity (DNT) (U.S. EPA (2001); UNEP (2002). Methylmercury is also the most common form of Hg to which humans are exposed (Berglund et al. 2005), and approximately 3% of the U.S. population is exposed to MeHg at concentrations above U.S. EPA recommended limits (Mortensen et al. 2014). Most human exposure to MeHg occurs via consumption of contaminated fish or seafood (Basu et al. 2018), but other foods also play a role in human exposure (Wells et al. 2020).

Concentrations of Hg (measured as total mercury (THg) or MeHg) in various biological matrices were employed as biomarkers of exposure to examine the association between MeHg and several adverse health effects, including DNT, in epidemiology studies. For DNT health effects, the most frequently used biomarkers of prenatal exposure are THg or MeHg measured in cord blood, maternal blood, and maternal hair. Although the current U.S. EPA reference dose (RfD) (U.S. EPA 2001) considered epidemiological studies using these three biomarkers, the quantitative estimate was based upon an epidemiological study using cord blood samples (NRC 2000). Because different cohorts have relied on different biomarkers and it is not clear which is a more sensitive measure of effects of MeHg exposure in the fetus, the question regarding which biomarkers are most appropriate for modeling DNT effects of MeHg has been debated for many years. While Cernichiari et al. (1995) recommended modeling using the maternal hair biomarker, others investigators suggested utilizing cord blood (Grandjean and Budtz-Jørgensen 2007; NRC 2000). The recent National Academies of Sciences, Engineering, and Medicine (2024) report indicated that cord blood Hg may be a better biomarker of fetal exposure than Hg in maternal hair.

To our knowledge, there exists no systematic comparison of the sensitivity of different biomarkers for estimating risk of DNT effects. Conducting such a comparison is challenging because most investigations employ only a single biomarker of exposure. In addition, for the same biomarker, different authors use various transformations of exposure including logarithms with different bases such as − 2, e, 10, making direct comparisons of reported modeling results across studies difficult. Dzierlenga, Crawford, and Longnecker (2020) developed statistical methods that enable re-expression of results obtained with log-transformed exposure to estimate the results that would be obtained from untransformed exposure which used this methodology in this review.

The aim of this study was to assess which biomarker of exposure is more sensitive for risks of DNT effects of MeHg, using data from investigations that collected more than one biomarker in the same population to facilitate direct comparison.

## Methods

### Literature review methods

The comprehensive literature search was designed to identify studies of MeHg exposure and DNT effects as part of a broader systematic review described elsewhere (U.S. EPA 2020). The first step was a literature search for the 1998–2022 period utilizing several databases including PubMed, Web of Science, Toxline, Science Direct, and SCOPUS. This time frame was selected to identify studies published after the peer review process for the 2001 US EPA assessment was well underway. From studies published before 1998, only one study from the Faroe Islands collected more than one biomarker, and these authors continued publishing after 1998. The literature search was conducted according to Populations, exposures, comparators, outcomes (PECO) criteria (Supplemental Table S2) that provide information regarding inclusion/exclusion criteria for the overall systematic review and this refined analysis. The main reason for study exclusion was a lack of dose-response information in humans. After removing duplicate references, literature search results were filtered by applying specifically developed epidemiology and dose-response search strings in SWIFT Review software (https://www.sciome.com/swift-review/). SWIFT Review enables custom search strings to filter title/abstracts of a set of studies. The resulting investigations were uploaded into DistillerSR software (https://www.distillersr.com/products/distillersr-systematic-review-software) for title/abstract and full-text screening by two independent reviewers to identify studies with quantitative dose-response data for MeHg DNT effects. DistillerSR enables several literature reviewers to screen both at title/abstract and full-text levels with a mechanism for conflict resolution. In the rare cases of a conflict between reviewers that could not be resolved between them, a third reviewer was involved in resolution.

Included studies were then moved forward to assess evaluation and consideration of study sensitivity and risk of bias using the Integrated Risk Information System (IRIS) approach (U.S. EPA 2022), which assesses domains of participant selection, exposure measurement, outcome ascertainment, confounding, analysis, selective reporting, and study sensitivity. Each investigation was evaluated by at least two independent reviewers. Study evaluations were documented in HAWC software (https://hawc.epa.gov/). EPA HAWC is an application that enables data and decisions supporting an assessment to be assessed and managed in modules (e.g., study evaluation, summary study data) that might be made publicly accessed online. Critical appraisal of risk of bias was important for this project in order to establish that differences in the observed magnitudes of effect between different exposure matrices were more likely explained by true differences in sensitivity of the biomarkers rather than bias. Only studies classified as *medium* or *high* confidence were considered for further analysis. By definition, any limitations identified in these studies are considered unlikely to have a significant impact on the study results.

Most sources of potential bias were not expected to differ between various exposure biomarkers within the same study (e.g., the DNT testing procedures did not depend upon the exposure biomarker used). However, there was potential for differences in the exposure measurement domain. Analytical chemistry methods for measurement of Hg biomarkers require specialized expertise and are subject to complications compared with analysis of other metals, such that these studies were examined for exposure measurement quality. The criteria used by the reviewers for evaluation of analytical chemistry methods are presented in Supplemental Materials, Supplemental Tables S3 and S4. Briefly, included studies, which received ratings of adequate or higher for the exposure measurement domain met or exceeded the following criteria: >50% of the samples were above the LOD; sample collection procedures were described, and for hair samples, the description included the site on the body and distance from that site where hair was cut; at no point during sample collection, preparation, or analysis did the samples exceed a temperature of 65°C potentially resulting in loss of sample analyte such as hair sample washing and drying, or open-vessel sample digestion. In addition, to attain a rating of adequate or higher, studies using standard validated methods such as EPA 7473 (THg) for blood (full list of standard validated methods are listed in the protocol) were required to have reported the results of at least one of the following QC steps confirming optimal performance of the method: (1) analysis of an appropriate standard or certified reference material, (2) a record of reliable performance in an interlab comparison program, (3) method recovery of 85% or higher for blood and 80% or higher for hair, (4) coefficient of variation of 15% or higher for blood and 25% or higher for hair. Studies utilizing non-standard methods (i.e., developed by the research team performing the study) were required to report at least two of the three QC steps listed above.

There were no *high* confidence studies. Investigations that were rated as *medium* confidence underwent full data extraction of results in Distiller SR. Extracted data included the following: demographics, exposure measures, including transformation of exposure and biomarkers of exposure, DNT tests and sub-tests with ages of administration and dose-response information (beta or BMD and its confidence interval). The extracted data from MeHg DNT studies were categorized based upon the tests and sub-tests of neuropsychological assessments modeled in the studies (e.g., motor domain sub-test of BSID III (Bayley 2006)). The subdivisions covered 7 domains of neurodevelopment including attention, executive function, motor function, learning and memory, verbal/language, social-emotional, and visuospatial function and 4 omnibus tests including general intelligence quotient (IQ), academic achievement, developmental, and neurophysiological assessment batteries. Details on the tests for each domain of neurodevelopment and for the omnibus tests were described by White et al. (2022), which also provides information on the quality of the tests. These domain classifications were used to group results for analysis.

## Statistical analysis

The effect estimates extracted from the epidemiology papers were those originally modeled by the study authors after log-transformation of exposure measures using different bases (such as 2, e, or 10). These modeling results including betas and their confidence intervals were re-expressed (Dzierlenga, Crawford, and Longnecker 2020) to the modeling results that might be obtained without the log-transformation of exposure to facilitate comparisons across studies.

Quantitative results extracted from the studies (betas and their confidence intervals as well as benchmark doses (BMDs) and their corresponding lower bounds (BMDLs)) were converted to the common maternal blood biomarker equivalents by employing standard conversion factors (1.7 for cord blood and 250 for maternal hair) (Grandjean and Budtz-Jørgensen 2007; Stern and Smith 2003).

After conversion, analyses were performed that were limited to only those studies that presented results for both maternal hair and cord/maternal blood biomarkers. These analyses were performed for each DNT domain and each study analyzing an endpoint in this domain such as Motor DNT domain and BSID III gross motor test. Summary statistics were calculated as the absolute value of % difference between study/endpoint results based upon maternal hair biomarkers and results from the same study/endpoint based upon cord blood/maternal blood biomarkers.

All the comparisons were made using confidence bounds on the regression coefficient. Selection of upper or lower confidence bound depended upon the adversity direction for a particular sub-test or test (i.e., lower confidence bound when adversity increases with lower DNT score values and upper confidence bound when adversity rises with higher DNT score values). The confidence bounds on regression coefficients were selected because they are usually used as a point of departure (POD) for estimating an RfD (U.S. EPA 2022). Effect size was used as a proxy for sensitivity as it indicates that the biomarker was better able to distinguish a true effect (assuming no differential bias, which is unlikely based upon the study evaluation approach). For multiple investigations that used the same participants, only one study was employed to avoid data duplication such as including original studies instead of a meta-analysis.

## Results

The literature search identified 17,661 studies. From these, after filtering for investigations with epidemiology and dose-response content and evaluation of risk of bias or study sensitivity, 7 papers remained that included modeling results for both cord blood and maternal hair exposure measurements. Two studies were excluded because these used the same subjects that were studied in other papers, leaving 5 studies for the analysis (Table 1).

**Figure 1.**
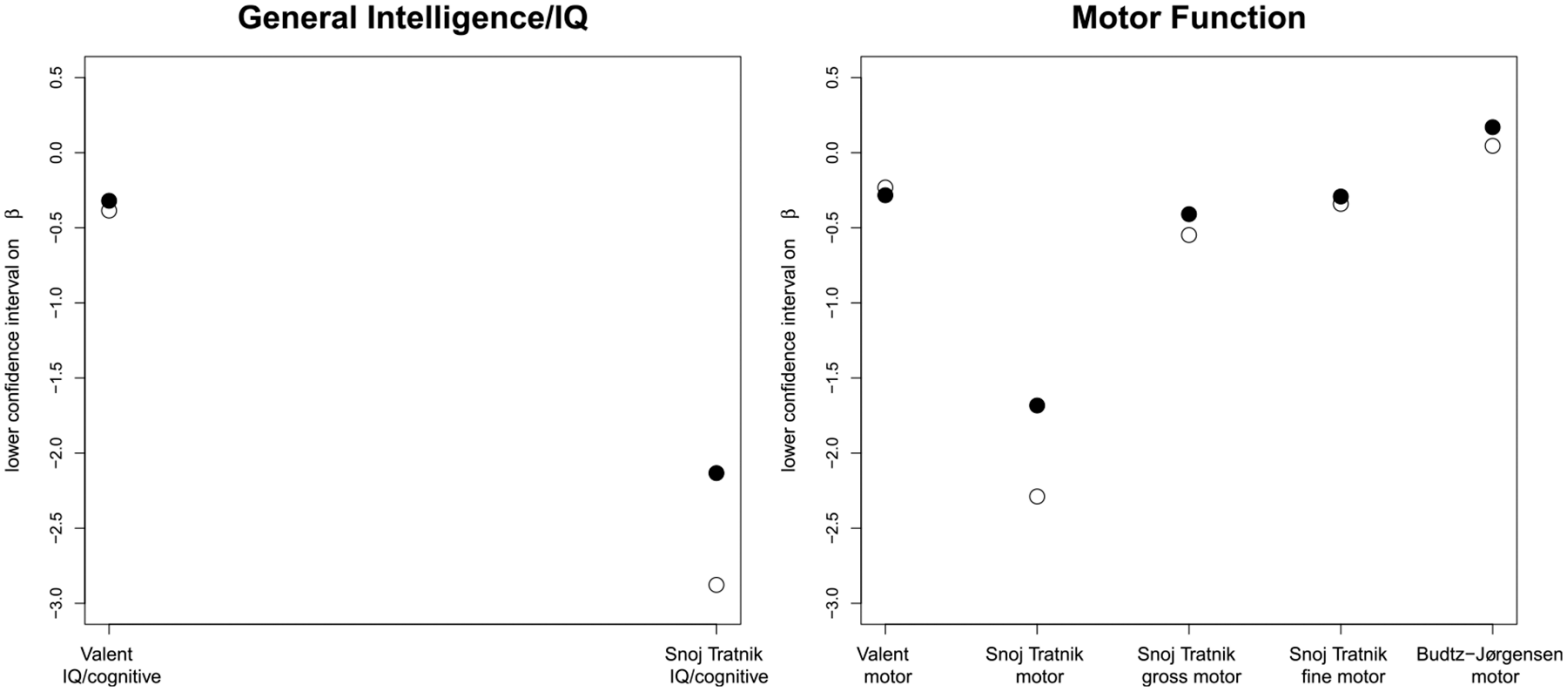
Comparison of lower confidence intervals on the regression coefficient (β) in the studies. For both domains of neurodevelopment, more adverse results are lower. Empty circles denote cord blood biomarker and filled circles denote maternal hair biomarker. X axes present study name and domain of neurodevelopment in the study.

**Figure 2.**
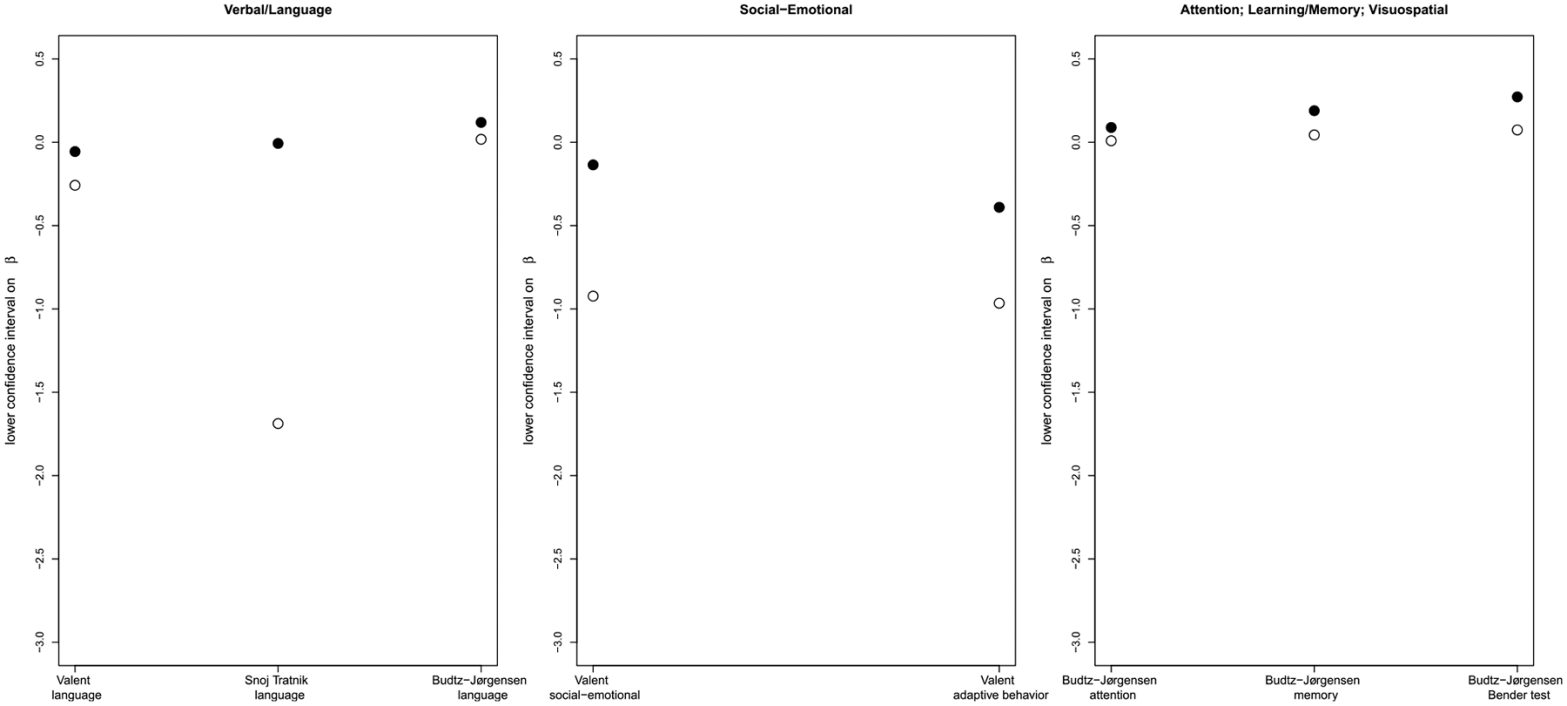
Comparison of lower confidence intervals on the regression coefficient (β) in the studies. For all 5 domains of neurodevelopment, more adverse results are lower. Empty circles denote cord blood biomarker and filled circles denote maternal hair biomarker. X axes provide study name and domain of neurodevelopment in the study. Budtz-Jørgensen et al (1999) BMD modeling results are divided by 100 to be on the same scale as beta from the other publications.

**Figure 3.**
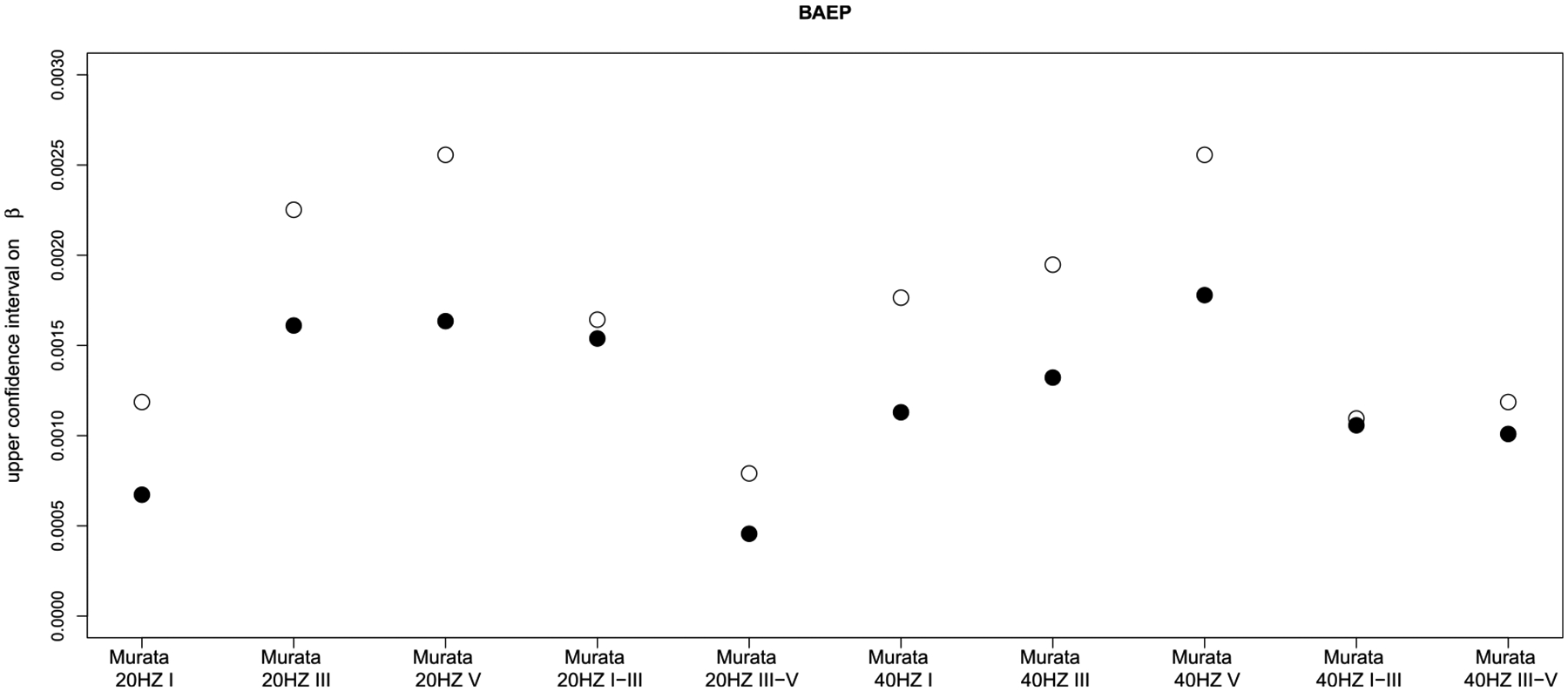
Comparison of upper confidence intervals on the regression coefficient (β) in the studies. For BAEP tests, more adverse results are higher. Empty circles denote cord blood biomarker and filled circles denote maternal hair biomarker. X axes provide study name and frequency of BAEP test.

**Figure 4.**
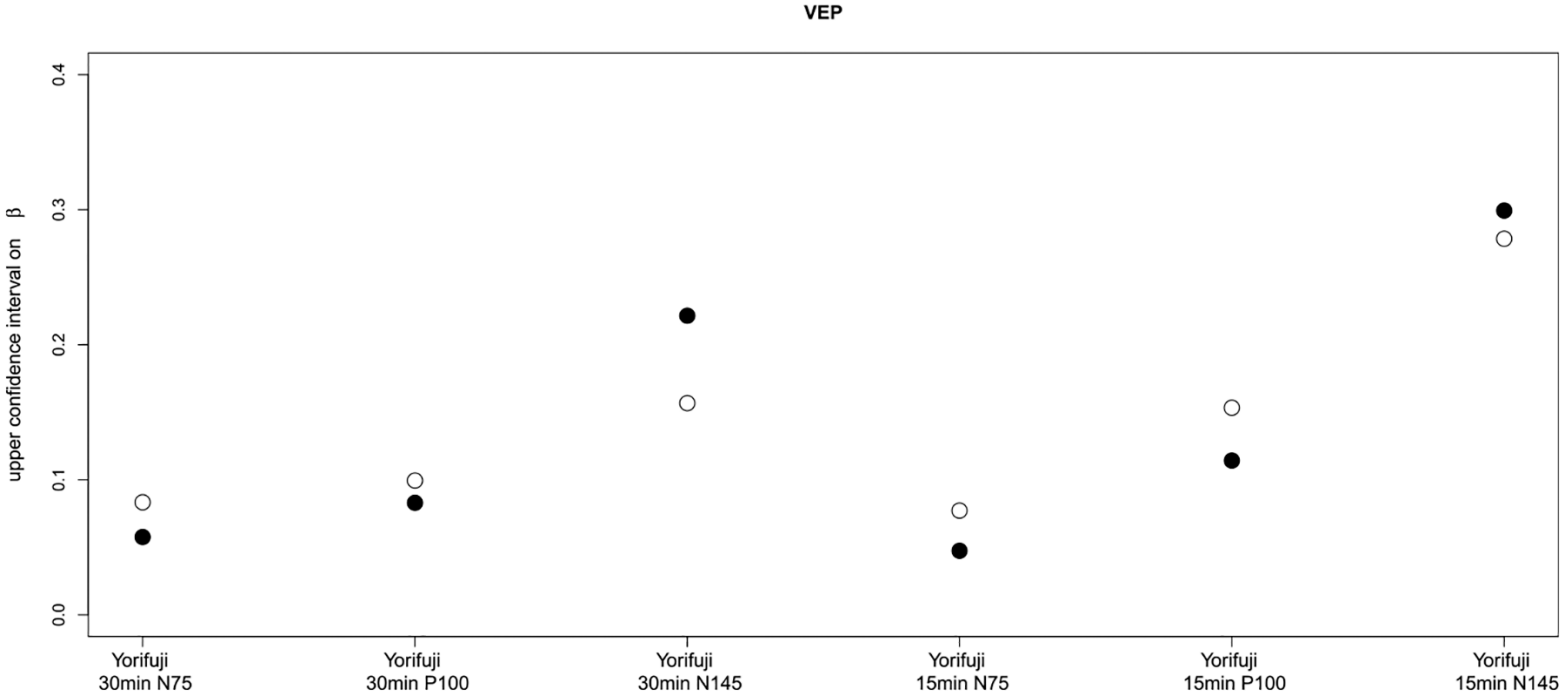
Comparison of upper confidence intervals (for VEP tests, more adverse results are higher) on the regression coefficient (β) in the studies. Empty circles denote cord blood biomarker and filled circles denote maternal hair biomarker. X axes present study name and latencies/peaks of VEP test.

The 5 papers considered in this analysis determined the effects of MeHg exposure on 7 domains of neurodevelopment (seen below in Figure 1–4). In comparing cord blood Hg and maternal hair Hg, cord blood Hg results were more sensitive than maternal hair Hg for every observation in 5 of the domains and results were mixed for the other two domains. Considering data from all domains together, cord blood Hg was more sensitive (larger effect estimate) in 32 out of 35 comparisons (Table 2). Further, when cord blood Hg modeling results displayed larger effects, these differed from hair Hg findings to a greater extent (mean 75%; range 4–583%) than in the cases where maternal hair Hg results showed larger effects (mean 18%; range: 7–29%). Even in the two domains where results were mixed depending upon test/subtest (motor and neuropsychological (VEP test)), the cord blood biomarker sensitivity (mean 43%; range 20–72% range) was greater than for the maternal hair biomarker (mean 18%, range 7–29%).

To illustrate these differences, results from two DNT domains are illustrated in Figure 1, one where cord blood Hg was always more sensitive (general intelligence/IQ domain measured in developmental test, left panel) and one where observations were mixed (motor domain, right panel). Figures 2–4 demonstrate the rest of the comparisons. Supplemental Table S1 provides data for the figures as well as original published results.

In the studies considered in this analysis, maternal hair was collected most frequently at birth (three studies) or during the third trimester of pregnancy (one study). Both time periods reflect late gestational exposure (Table 1). The single investigation that collected hair in the second trimester was the only study in which hair Hg was more sensitive than cord blood Hg for the motor domain.

There were two studies (Barbone et al. 2019; Rothenberg et al. 2016) that used maternal hair Hg and maternal blood Hg as MeHg biomarkers (maternal blood Hg was more sensitive: mean 320%; range 43%−855%, based upon 7 comparisons (unadjusted comparison only was available in Rothenberg et al. 2016)). In addition, two studies (Barbone et al. 2019; Kim et al. 2020 (there also was Ryu et al. (2017) study of the same children)) of cord blood Hg and maternal blood Hg (maternal blood Hg more sensitive: mean 36%, range 15%−71%) based upon 10 comparisons, and in two comparisons, cord blood Hg was more sensitive (3% and 13%).

## Discussion

The comparisons described here demonstrated that cord blood Hg biomarker typically tends to be associated with more sensitive results than maternal hair Hg biomarker for modeling of MeHg DNT effects across most DNT domains. One possible explanation for this observation is that the analytical chemistry accuracy and precision in determining MeHg levels are generally less variable for cord blood samples (e.g., smaller coefficient of variation for replicate samples) than these are for maternal hair samples due to factors specific to the collection, preparation, and analysis of hair (U.S. EPA 2020). For this reason, blood Hg studies were required to demonstrate a higher level of method precision as compared to hair Hg investigations to be considered for the current analysis. Most epidemiology studies based upon biomarkers of exposure are likely to possess some amount of nondifferential exposure measurement error and misclassification, which is expected to bias the results toward the null on average, but the magnitude of misclassification may differ by biomarker type. Assuming minimal bias away from the null, cord blood Hg being a more sensitive biomarker may be explained by this greater precision.

Another possible explanation is that cord blood Hg may better represent the level of MeHg in the fetal compartment than hair does. This is consistent with the NASEM report (2024) suggestion that cord blood Hg may be a more reliable biomarker of fetal exposure than Hg in maternal hair. Although one potential benefit of hair as a biomarker is the ability to capture a longer period of exposure and act as an average over regular but infrequent exposures to MeHg. It appears that the indirectness of this measure in representing fetal exposure, the greater is the analytical variability in hair measurement. Further the high frequency of fish consumption (and thus MeHg exposure) in the cohorts of interest thus outweigh this potential benefit. The high frequency of exposure might result in hair and blood levels that are at steady state, as clearance of MeHg is relatively slow, with a half-life of 50 days in blood Rand and Caito (2019). Therefore, cord blood Hg may represent, not just recent exposures, but exposures going back much longer.

There were only two studies each that enabled comparison of maternal blood and maternal hair or cord blood. While limited, data indicated that maternal blood is also preferable to maternal hair and also to cord blood, although more data is needed to make conclusions regarding maternal blood.

Although other biomarkers such as cord tissue, placenta tissue, dry blood spots, and toenails can be considered, these biomarkers are much less frequently examined in modeling of DNT effects in epidemiological studies and, therefore, it was not possible to make comparisons. While the 2001 US EPA RfD was calculated based upon a prenatal exposure effect, it is not clear at this point whether the prenatal period represents the only critical window for MeHg DNT effects. Therefore, including childhood postnatal exposures in this analysis was considered. However, the lack of a reliable approach to convert children’s biomarkers to maternal or cord blood biomarkers prevented incorporation of children’s biomarkers into the current analysis. This is a limitation, as children’s MeHg exposures diverge from maternal/fetal exposures that reflect *in utero* exposures, as early as at one year of age (Grandjean et al. 1997). Another limitation of this analysis is that only DNT effects were assessed, and it is unclear which biomarker is better for modeling of other effects of MeHg such as cardiotoxicity in adults or for DNT effects of other chemicals.

All the studies in this analysis used THg as a surrogate of MeHg exposure in reported DNT modeling results due to lack of direct measurements of MeHg. Valent et al. (2013) modeled a nonrandom subset of the cohort with MeHg measurements in hair and cord blood, but data were not shown. At the exposure levels in studies analyzed here (Table 1), THg was found to be a reliable predictor of MeHg in both maternal hair and blood (Valent et al. 2013; Wells et al. 2022; National Academies of Sciences, Engineering, and Medicine [NASEM] 2024).

Although all studies included in this analysis received medium confidence in the systematic review, this is not a significant limitation. Requirements for high-confidence studies are stringent and medium-confidence study results are not expected to exert substantial bias. The risk of bias evaluation included a careful evaluation of confounding factors. The medium-confidence overall rating which required a medium or high confidence rating for the confounding domain implies that confounding adjustment was appropriate for the study/endpoint and that the potential confounders most likely to bias results were adjusted for. Because hair and blood modeling results are compared within each study/endpoint, adjustment for confounding performed by the original investigators was equivalent for hair and blood for each comparison. Thus, confounding is not expected to influence the results of this analysis.

The exposure levels in the studies varied (Table 1), and the 2 studies with mixed sensitivity results for the comparison of maternal hair and cord blood did not occur at extreme exposure ranges compared to investigations that showed cord blood to be more sensitive across all test/sub-tests. Because each comparison is within a study and endpoint, each comparison possesses an equivalent exposure level.

This analysis used a methodology (Dzierlenga, Crawford, and Longnecker 2020) for re-expression that provides an estimate of the results of a model if exposure were not log-transformed from the published findings of a model with log-transformed exposure. Recently, Linakis et al. (2024) compared the methodology used here with two other re-expression methodologies and concluded that in some situations bias with respect to truth may be 20–25%. However, that analysis does not inform the direction of possible bias that may either slightly exacerbate or reduce differences when re-expressed results are compared. Still, this possible bias presents a limitation for this study that might be avoided if investigators commonly provided results using exposure that was not log-transformed.

Because collecting an array of biomarkers simultaneously for an epidemiological study is expensive, it is not surprising that only cohorts with multiple publications (i.e., PHIME (Public health impact of long term, low-level mixed element exposure in susceptible population strata) and two Faroe Islands cohorts) provided data that enabled our comparison. Noting this limitation, the populations in this analysis still represented variations in diet and location (4 countries), age of children tested (1.5–14 years), and covered almost all domains of DNT. Therefore, there is a great potential for our findings to be generalized to other cohorts.

Hair biomarkers still are useful for surveillance or research purposes. For instance, hair is both less expensive and less invasive than blood to collect and store. However, there is potential exposure misclassification when using hair biomarker measurements because of some hair treatments that also contain Hg (Dakeishi et al. 2005). Therefore, THg or MeHg in hair remains an appropriate biomarker for some population-level MeHg exposure analyses, such as identifying populations of concern and characterizing long-term temporal trends in MeHg exposures for many populations. However, cord blood biomarkers are more sensitive and are preferable to maternal hair biomarkers for dose-response modeling of DNT effects of MeHg.

## Supplementary Material

Supplement1

## Figures and Tables

**Table 1.** Summary of the studies providing both cord blood and maternal hair biomarker modeling results in the same study.

Author, Year	Location	N	Child age (y)	Cord blood exposure (μg/L) *	Maternal hair exposure (μg/g) *	Maternal hair timing	Maternal Hair length/location	DNT Tests
Studies used in the analysis								
Budtz-Jørgensen et al. (1999)	Faroe Islands Cohort I	917	7	GM 22.9 IQR (13.4–41.3)	GM 4.27 IQR (2.6–7.7)	Birth	at least 100 mg, most were more than 5 cm in length, close to the root	NES2, BNT, Bender, CVLT
Murata et al. (2004)	Faroe Islands Cohort I	859	14	GM 22.6 IQR (13.2–40.8)	GM 4.2 IQR (2.55–7.68)	Birth	at least 100 mg, most were more than 5 cm in length, close to the root	BAEP
Snoj Tratnik et al. (2017)	Croatia, Slovenia	361	1.5	GM 2.05 95% CI 2.05 (1.87–2.25)	GM 0.36 95% CI (0.33–0.40)	34 week or birth	1–3 cm closest to scalp	BSID III
Valent et al. (2013)	Italy	606	1.5	Mean (SD) 5.54 (4.83) IQR (2.40–7.02)	Mean (SD) 1.06 (1.03) IQR (1.03–1.28)	20–22 week	1 gram	BSID III
Yorifuji et al. (2013)	Faroe Islands Cohort II	139	7	GM 22.8 IQR (13.7 – 41.2)	GM 4.6 IQR (2.7 – 8.2)	Birth	at least 100 mg, most were more than 5 cm in length, close to the root	VEP
Studies not used in the analysis								
Barbone et al. (2019) (used same subjects as Snoj Tratnik et al. (2017) and Valent et al. (2013))	Croatia, Greece, Slovenia, Italy	1200	1.5	Mean (SD) 5.51 (5.0) Range 0.1–39.6	Mean (SD) 1.0 (1.0) Range 0.017–13.5	From 20 week to birth	Varies from 1–3 cm to 1 gram, close to scalp	BSID III
Murata et al. (1999) (used same subjects as Murata et al. (2004)	Faroe Islands Cohort I	388	7	GM 22.6 IQR (13.2–40.8)	GM 4.2 IQR (2.55–7.68)	Birth	at least 100 mg, most were more than 5 cm in length, close to the root	BAEP

* Units converted to the same units (assuming μg/L=ng/g for cord blood).

Abbreviations/References: GM – geometric mean, SD – standard deviation, CI – confidence interval, IQR – inter-quartile range (25%−75%), BAEP – Brain stem auditory evoked potentials (Araki and Murata 1993), Bender – Bender Gestalt Test (Schlange et al. 1977), BNT – Boston Naming Test (Kaplan, Goodglass, and Weintraub 1983), BSID- Bayley Scales of Infant and Toddler Development (Bayley 2006), CVLT – California Verbal Learning Test (Children) (Delix et al. 1994), NES2 – Neurobehavioral Evaluation System (NES) (Letz and Baker 1988), VEP -Visual evoked potentials (Araki and Murata 1993).

**Table 2.** Comparing sensitivity of modeling results of cord blood and maternal hair biomarkers. The difference between the appropriate (depending on the direction of adversity) confidence bounds on the regression coefficient (β) in the studies. There are more comparisons listed in table 2 than tests in table 1 because table 2 includes subtests, whereas table 1 does not. Budtz-Jørgensen et al. (1999) applied 4 different statistical models but here only the best fitting model (with BMR = 0.05) is reported. Other studies applied only one statistical model.

Study	Number of subtests with cord blood more sensitive than maternal hair/total number of subtests	Range (mean; median) of relative difference when cord blood is more sensitive	Range (mean; median) of relative difference when maternal hair is more sensitive
Budtz-Jørgensen et al. (1999)	5/5		
Murata et al. (2004)	8/8		
Snoj Tratnik et al. (2017)	5/5		
Valent et al. (2013)	4/5		
Yorifuji et al. (2013)	10/12		
Overall	32 (35)	4–583% (75%; 44%)	7–29% (18%; 18%)
