# Supplementary material for: Which prenatal biomarker is most appropriate for methylmercury dose-response for neurodevelopmental effects?
Source: J Toxicol Environ Health B Crit Rev. Author manuscript; Available in PMC 2026 May 19. (PMC11961308; doi:10.1080/10937404.2024.2444650)
Supplement: Supplement1 [file NIHMS2053121-supplement-Supplement1.docx]

Supplemental materials content:

Supplemental Table 1 Details of the published and converted data from the studies that presented both cord blood and maternal hair measurements and that were used in the analysis.

Supplemental Table 2. Overall systematic review populations, exposures, comparators, outcomes (PECO) criteria for epidemiology dose-response data.

Supplemental Table 3. Evaluation of analytical methods for blood total and methylmercury in epidemiology studies

Supplemental Table 4. Evaluation of analytical methods for hair total and methylmercury in epidemiology studies

| Author, Year, Test | Subtest | Biomarker | N | Published results | | | | Re-expressed results and  converted to ng/g maternal blood | | |
| --- | --- | --- | --- | --- | --- | --- | --- | --- | --- | --- |
|  |  |  |  | Beta or BMD5 | CI lower bound or BMDL5 | CI upper bound | Units | Beta or BMD5 | CI lower bound or BMDL5 | CI upper bound |
| Lower modeling results are associated with a greater DNT risk | | | | | | | | | | |
| Valent  et al. 2013  BSID III | Cognitive | Maternal Hair | 505 | -2.2 E-03 | -1.04 | 1.04 | ng/g | -6.14 E-04 | -0.32 | 0.32 |
|  |  | Cord Blood | 378 | 5.2 E-02 | -1.00 | 1.10 | ng/g | 2.0 E-02 | -0.38 | 0.42 |
|  | Language | Maternal Hair | 505 | 0.85 | -0.18 | 1.87 | ng/g | 0.26 | -5.52 E-02 | 0.58 |
|  |  | Cord Blood | 378 | 0.41 | -0.67 | 1.50 | ng/g | 0.16 | -0.26 | 0.58 |
|  | Motor | Maternal Hair | 505 | -0.19 | -0.92 | 0.55 | ng/g | -5.77 E-02 | -0.28 | 0.17 |
|  |  | Cord Blood | 378 | 0.16 | -0.60 | 0.91 | ng/g | 6.00 E-02 | -0.23 | 0.35 |
|  | Social-Emotional | Maternal Hair | 362 | 1.77 | -0.44 | 3.89 | ng/g | 0.54 | -0.14 | 1.19 |
|  |  | Cord Blood | 271 | -7.1 E-02 | -2.40 | 2.26 | ng/g | -2.73 E-02 | -0.92 | 0.87 |
|  | Adaptive Behavior | Maternal Hair | 362 | 0.55 | -1.27 | 2.36 | ng/g | 0.17 | -0.39 | 0.72 |
|  |  | Cord Blood | 271 | -0.57 | -2.51 | 1.37 | ng/g | -0.22 | -0.96 | 0.53 |
| Snoj Tratnik et al. 2015  BSID III | Cognitive | Maternal Hair | 280 | -1.12 | -3.08 | 0.84 | ng/g | -0.78 | -2.13 | 0.58 |
|  |  | Cord Blood | 283 | -1.4 | -3.47 | 0.66 | ng/g | -1.17 | -2.88 | 0.55 |
|  | Language | Maternal Hair | 280 | -1.0 E-02 | -2.32 | 2.30 | ng/g | -6.92 E-03 | -1.61 | 1.59 |
|  |  | Cord Blood | 283 | -1.03 | -3.46 | 1.40 | ng/g | -0.85 | -2.87 | 1.16 |
|  | Motor | Maternal Hair | 280 | -1.04 | -2.43 | 0.34 | ng/g | -0.72 | -1.68 | 0.24 |
|  |  | Cord Blood | 283 | -1.16 | -2.76 | 0.44 | ng/g | -0.96 | -2.29 | 0.36 |
|  | Fine Motor | Maternal Hair | 280 | -0.29 | -0.59 | 0.01 | ng/g | -0.20 | -0.41 | 6.92 E-03 |
|  |  | Cord Blood | 283 | -0.33 | -0.66 | -1.0 E-02 | ng/g | -0.27 | -0.55 | -8.29 E-03 |
|  | Gross Motor | Maternal Hair | 280 | -0.12 | -0.42 | 0.18 | ng/g | -8.31 E-02 | -0.29 | 0.12 |
|  |  | Cord Blood | 283 | -0.10 | -0.41 | 0.22 | ng/g | -8.29E-02 | -0.34 | 0.18 |
| Budtz-Jørgensen et. al. 1999  NES2 | Motor | Maternal Hair | 914 | 18.74 | 4.27 | NR | µg/g | 74.96 | 17.08 | NR |
|  |  | Cord Blood | 894 | 51.6 | 7.92 | NR | µg/L | 30.35 | 4.66 | NR |
|  | Attention | Maternal Hair | 914 | 7.43 | 2.23 | NR | µg/g | 29.72 | 8.92 | NR |
|  |  | Cord Blood | 894 | 3.03 | 1.6 | NR | µg/L | 1.78 | 0.94 | NR |
| Budtz-Jørgensen et. al. 1999  BNT | With cues | Maternal Hair | 914 | 8.35 | 2.99 | NR | µg/g | 33.4 | 11.96 | NR |
|  |  | Cord Blood | 894 | 6.46 | 3.11 | NR | µg/L | 3.80 | 1.83 | NR |
| Budtz-Jørgensen et. al. 1999  Bender | Vision | Maternal Hair | 914 | 91.57 | 6.82 | NR | µg/g | 366.28 | 27.28 | NR |
|  |  | Cord Blood | 894 | 270.82 | 12.66 | NR | µg/L | 159.31 | 7.45 | NR |
| Budtz-Jørgensen et. al. 1999  CVLT | Memory | Maternal Hair | 914 | 27.16 | 4.75 | NR | µg/g | 108.64 | 19.0 | NR |
|  |  | Cord Blood | 894 | 49.51 | 7.56 | NR | µg/L | 29.12 | 4.45 | NR |
| Higher modeling results are associated with a greater DNT risk | | | | | | | | | | |
| Yorifuji  et al. 2013  VEP | 30 min  N75 | Maternal Hair | 138 | 0.23 | -0.33 | 0.79 | µg/L | 3.22 E-02 | -1.91 E-02 | 8.35 E-02 |
|  |  | Cord Blood | 138 | 0.32 | -0.19 | 0.83 | µg/g | 1.68 E-02 | -2.41 E-02 | 5.78 E-02 |
|  | 30 min P100 | Maternal Hair | 138 | 0.25 | -0.64 | 1.14 | µg/L | 2.01 E-02 | -5.93 E-02 | 9.96 E-02 |
|  |  | Cord Blood | 138 | 0.20 | -0.59 | 0.99 | µg/g | 1.83 E-02 | -4.65 E-02 | 8.31 E-02 |
|  | 30 min  N145 | Maternal Hair | 138 | 1.09 | -0.85 | 3.03 | µg/L | -1.31 E-02 | -0.18 | 0.16 |
|  |  | Cord Blood | 138 | -0.13 | -1.82 | 1.56 | µg/g | 7.98 E-02 | -6.20 E-02 | 0.22 |
|  | 15 min  N75 | Maternal Hair | 139 | −8.0 E-02 | -0.81 | 0.65 | µg/L | 1.31 E-02 | -5.12 E-02 | 7.73 E-02 |
|  |  | Cord Blood | 139 | 0.13 | -0.51 | 0.76 | µg/g | -5.86 E-03 | -5.93 E-02 | 4.76 E-02 |
|  | 15 min  P100 | Maternal Hair | 139 | 0.57 | -0.42 | 1.56 | µg/L | 6.24 E-02 | -2.92 E-02 | 0.15 |
|  |  | Cord Blood | 139 | 0.62 | -0.29 | 1.52 | µg/g | 4.17 E-02 | -3.09 E-02 | 0.11 |
|  | 15 min  N145 | Maternal Hair | 139 | 2.22 | 0.35 | 4.09 | µg/L | 0.10 | -7.54 E-02 | 0.28 |
|  |  | Cord Blood | 139 | 1.01 | -0.75 | 2.77 | µg/g | 0.16 | 2.56 E-02 | 0.30 |
| Murata  et. al. 2004  BAEP | BAEP 20HZ I | Maternal Hair | 855 | 1.0 E-03 | -2.6 E-02 | 2.8 E-02 | µg/L | 4.57 E-04 | -2.74 E-04 | 1.19 E-03 |
|  |  | Cord Blood | 835 | 1.5 E-02 | -9.0 E-03 | 3.9 E-02 | µg/g | 2.40 E-05 | -6.25 E-04 | 6.73 E-04 |
|  | BAEP 20HZ III | Maternal Hair | 855 | 3.7 E-2 | 7.0 E-03 | 6.7 E-02 | µg/L | 1.37 E-03 | 4.87 E-04 | 2.25 E-03 |
|  |  | Cord Blood | 835 | 4.5 E-02 | 1.6 E-02 | 7.4 E-02 | µg/g | 8.90 E-04 | 1.68 E-04 | 1.61 E-03 |
|  | BAEP 20HZ V | Maternal Hair | 855 | 3.3 E-02 | -4.0 E-03 | 6.8 E-02 | µg/L | 1.49 E-03 | 4.26 E-4 | 2.56 E-03 |
|  |  | Cord Blood | 835 | 4.9 E-02 | 1.4 E-02 | 8.4 E-02 | µg/g | 7.70 E-04 | -9.62 E-05 | 1.64 E-03 |
|  | BAEP 20HZ I-III | Maternal Hair | 855 | 3.6 E-02 | 8.0 E-03 | 6.4 E-02 | µg/L | 8.22 E-04 | 0 | 1.64 E-03 |
|  |  | Cord Blood | 835 | 2.7 E-02 | -1.0 E-04 | 5.4 E-02 | µg/g | 8.66 E-04 | 1.92 E-04 | 1.54 E-03 |
|  | BAEP 20HZ III-V | Maternal Hair | 855 | -5.0 E-03 | -2.9 E-02 | 1.9 E-02 | µg/L | 1.22 E-04 | -5.48 E-04 | 7.91 E-04 |
|  |  | Cord Blood | 835 | 4.0 E-03 | -1.8 E-02 | 2.6 E-02 | µg/g | -1.20 E-04 | -6.97 E-04 | 4.57 E-04 |
|  | BAEP  40HZ I | Maternal Hair | 855 | 1.4 E-02 | -1.9 E-02 | 4.7 E-02 | µg/L | 8.22 E-04 | -1.22 E-04 | 1.77 E-03 |
|  |  | Cord Blood | 835 | 2.7 E-02 | -4.0 E-03 | 5.8 E-02 | µg/g | 3.37 E-04 | -4.57 E-04 | 1.13 E-03 |
|  | BAEP 40HZ III | Maternal Hair | 855 | 2.3 E-02 | -1.0 E-02 | 5.5 E-02 | µg/L | 9.74 E-04 | 0 | 1.95 E-03 |
|  |  | Cord Blood | 835 | 3.2 E-02 | 3.0 E-4 | 6.4 E-02 | µg/g | 5.53 E-04 | -2.40 E-04 | 1.32 E-03 |
|  | BAEP 40HZ V | Maternal Hair | 855 | 3.6 E-02 | -2.0 E-03 | 7.4E-02 | µg/L | 1.46 E-03 | 3.6 E-04 | 2.56 E-03 |
|  |  | Cord Blood | 835 | 4.8 E-02 | 1.2 E-02 | 8.4 E-02 | µg/g | 8.66 E-04 | -4.81E-05 | 1.78 E-03 |
|  | BAEP 40HZ I-III | Maternal Hair | 855 | 9.0 E-03 | -2.6 E-02 | 4.4 E-02 | µg/L | 1.22 E-04 | -8.52 E-04 | 1.10 E-03 |
|  |  | Cord Blood | 835 | 4.0 E-03 | -2.8 E-02 | 3.6 E-02 | µg/g | 2.16 E-04 | -6.25 E-04 | 1.06 E-03 |
|  | BAEP 40HZ III-V | Maternal Hair | 855 | 1.3 E-02 | -1.6 E-02 | 4.2 E-02 | µg/L | 4.57 E-04 | -2.74 E-04 | 1.19 E-03 |
|  |  | Cord Blood | 835 | 1.5 E-02 | -9.0 E-03 | 3.9 E-02 | µg/g | 3.13 E-04 | -3.85 E-04 | 1.01 E-03 |
|  |  | Cord Blood | 894 | 270.82 | 12.66 | NR | µg/L | 159.31 | 7.45 | NR |

Supplemental Table 1 Details of the published and converted data from the studies that presented both cord blood and maternal hair measurements and that were used in the analysis. After re-expression (described in the main test) conversion factors were 1.7 from cord blood to maternal blood and 250 from maternal hair to maternal blood. Lesser number of significant digits than used is shown for table readability. Whenever (beta, exact p-value) were originally reported, they were converted to confidence interval assuming normality.

Abbreviations: CI – confidence interval, BMD5 – benchmark dose corresponding to 5% extra risk, BMDL5 – lower 95% confidence bound on benchmark dose, NR – not reported. For test abbreviations and citations see Table 1 legend in the main body. For Budtz-Jørgensen et. al. 1999 BMD5 and BMDL5 are from best-fitting (logarithmic) dose-response model.

| PECO element | Evidence |
| --- | --- |
| Populations | Human populations exposed during life stages ranging from the fetus through adolescence. |
| Exposures | Any quantitative exposure to MeHg based on biomonitoring data in hair and blood. Measurements must be either direct MeHg measurements or measurements of total mercury (not other forms of mercury, e.g., mercury salts). |
| Comparators | Referent populations exposed to lower (within the study) levels of MeHg will be used to examine specific effects. The results of the comparisons must be presented with sufficient detail of quantitative modeling (e.g., regression coefficients presented with statistical measure of variation).  Both maternal hair and cord or maternal blood results for the same outcome should be presented. |
| Outcomes | DNT outcomes measured at any age including, but not limited to, tests or measures of cognition, motor function, behavior, vision, and hearing. |

Supplemental Table 2. Overall systematic review populations, exposures, comparators, outcomes (PECO) criteria for epidemiology dose-response data.

The additional criteria used in this publication for Comparators are bolded.

| Level | | Criteria |
| --- | --- | --- |
| Good | 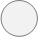 | Acceptable proportion of samples (>50%) above the limit of detection (LOD) OR LOD less than median blood mercury concentration in the study population  Method  All papers must include a description of methodological factors:   - All papers must indicate the type of sample analyzed; for Good quality, should be liquid whole blood (collected in tubes with ethylenediaminetetraacetic acid [EDTA] anticoagulant, stored in the refrigerator within 1 year) or a freeze-dried (within 2 years) blood sample.   NOTE: If samples have been stored for longer than noted above, recovery of storage stability spiked samples must be reported but results will not be evaluated. Additional information regarding storage stability samples is provided in “Useful Terms Defined.”   - For laboratories using a standard method: The paper will include the method number and a citation for a standard method for measuring mercury levels in blood (e.g., Centers for Disease Control and Prevention method ITB003A or National Health and Nutrition Examination Survey methods 3001.1 or 3016.8 for total mercury, method DLS-3020.5 for methylmercury) OR a description of the method discussed as follows. - For laboratories using a nonstandard, validated method: The paper will provide a citation for a peer-reviewed report of the validation in the main body of the paper or in supplementary information (refer to “Useful Terms Defined” for more information on validation) OR a description of the method discussed, as follows. - For laboratories using nonstandard, nonvalidated methods: Nonstandard methods may perform acceptably if sufficient evidence of data quality (quality control [QC] results) is provided (as follows). For nonstandard methods, the paper must provide a description of the method, including the following:   - Analytical limits including LOD and/or the limit of quantitation (LOQ) must be reported for nonstandard, nonvalidated methods to obtain a Good rating.   - Combustion Atomic Absorption Spectroscopy (CAAS) refers to direct mercury analysis and may use several instruments, such as the Milestone Direct Mercury Analyzer (DMA) or the Nippon Mercury Analyzer (MA). - For studies using CAAS for total mercury analysis, critical method variables that should be noted are time and temperature of sample drying step, charring/decomposition step of analysis, and amalgamator flash time and temperature.   - Sample stabilization measures (e.g., addition of gold or thiols; refer to “Useful Terms Defined” for additional details).   - Sample preparation method including, but not limited to, the following:     - Derivatization steps for gas chromatography-inductively coupled plasma-mass spectrometry (GC-ICP-MS) analysis or another speciation technique for methylmercury.     - Oxidizing agents for cold vapor atomic fluorescence spectroscopy (CVAFS) and cold vapor atomic absorption spectroscopy (CVAAS) (e.g., potassium permanganate [KMnO_4_], nitric acid, or sulfuric acid for total mercury analysis).     - Reducing agent for cold vapor atomic fluorescence spectroscopy (CVAFS) and cold vapor atomic absorption spectroscopy (CVAAS) (e.g., tin chloride [SnCl_2_], sodium borohydride [NaBH_4_], sodium hypochlorite [NaClO]).     - Digestion reagents, temperatures (≤65°C), and times.     - Extraction solvent composition. - For speciation analysis (i.e., methylmercury): Separation steps before detection. Examples include the following:   - For liquid chromatography (LC; e.g., high-performance liquid chromatography [HPLC] or ultra-high-performance liquid chromatography [UHPLC]): Column type, eluent composition (e.g., mercaptoethanol, EDTA, cysteine), eluent gradient (or indicate whether an isocratic program [where the same eluent content was used through the run] was used), and injection volume.   - For gas chromatography: Transfer line temperature, column temperature program, and column identity.   - For distillation: Solvent and temperature. - Use of an appropriate internal standard (e.g., bismuth [Bi], praseodymium [Pr], holmium [Ho]; ICP-MS only; other organomercury compound [e.g., propylmercury, butylmercury; speciation by LC- or GC-ICP-MS only]):   - NOTE: Internal standard is not generally included in CVAAS/CVAFS/CAAS methods.   NOTE: The items listed earlier should be included, but because of the great variability in nonstandard methods, it is not necessary to evaluate the quality of all methodological factors, only to ensure that they are included in the method description.  Quality Control  All papers (standard methods, validated methods, and nonstandard methods) must include a description of QC procedures and results performed to verify method performance and data quality for the study:   - At least three laboratory QC procedures and results in the methods section, supplementary materials, cited papers, or standardized laboratory protocol, including, but not limited to, the following:   - Blood-based standard reference materials (SRMs) (955C, levels 1 and 3 only for total mercury, level 3 for methylmercury only; 966 if the study was done before December 2015, level 2 for methylmercury only): % recovery information, 90%–110%. If the study was done before 2000 (the year SRM 966 was issued), acceptable reference materials include “Control Blood for Metals” by the Behring Institute, OSSD 20/21.   - Duplicate sample preparation and relative percent difference (RPD) results, <15% RPD.   - Representative blank sample analyses or chromatograms to demonstrate the absence of interferences.   - Recovery of spiked study samples, 90%–110%.   - Post-extraction spiked sample recovery or method blank spiked (MBS) samples, 90%–110%.   - Replicate QC precision (also called uncertainty, repeatability, or reproducibility; or percent relative standard deviation [%RSD] or coefficient of variation [%CV], <15%, or correlation coefficient, >0.90).   - Participation in an interlaboratory testing program with documented results for mercury in blood samples and “satisfactory” results.   NOTE: A wide range of interlaboratory testing programs is available for trace metals, but only ones that monitor mercury in whole blood, serum, or plasma, or that use standard methods, are relevant for assessing data quality.   - - Incurred sample reanalysis (ISR) to demonstrate reproducibility on different days, and RPD results, <15% RPD; refer to “Useful Terms Defined” for more information.   - Control charts (e.g., Bland-Altman, Levey-Jennings, Harrell-Davis, Shewhart) for QC samples of the same sample type, prepared by the cited method, that show method performance over time (large sample populations only).   NOTE: If any of the above method parameters or quality control measures exhibit values that fall outside the range of value noted above, then the entire study should be categorized at the lowest quality level of the individual method parameter(s) or QC measure(s). |
| Adequate | 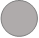 | Method  Same as Good, with the following exceptions:   - All papers must indicate the type of sample analyzed; for Adequate quality, may be liquid whole blood (collected in tubes with ethylenediaminetetraacetic acid [EDTA] anticoagulant, stored in the refrigerator within 1 year) or a freeze-dried (within 2 years) blood sample.   NOTE: If samples have been stored for longer than noted above, recovery of storage stability spiked samples must be reported but results will not be evaluated.   - For laboratories using standard methods: Samples analyzed by Environmental Protection Agency (EPA) method 7473 (total mercury only). - For nonstandard, nonvalidated methods: The paper will comment on two or three methodological details listed earlier such as extraction times, cleaning times between sample analyses, instrumental technique, and so on. - Analytical limits including LOD and/or the limit of quantitation (LOQ) must be reported for nonstandard, nonvalidated methods to obtain an Acceptable rating. - For measurements of methylmercury: Determined methylmercury by subtracting inorganic mercury from total mercury. - For ICP-MS only: Use of less common or less appropriate internal standards such as terbium (Tb), rhodium (Rh), gallium (Ga), thallium (Tl), indium (In), yttrium (Y), or scandium (Sc).   Quality Control  Any Adequate analysis must include the following:   - For standard methods and validated methods (with literature citation): Discussion of one or two laboratory QC procedures and results in the methods section, supplementary materials, cited papers, or standardized laboratory protocol, including either of the following:   - Reference material percentage recovery information (may be the National Institute of Standards and Technology [NIST] SRM mentioned earlier or another such as NIST 3133 or 3177 for total mercury only, or commercial blood SRM [e.g., ClinCheck or Seronorm for total mercury]); recovery will fall in the range 85%–90% or 110%–115%.   - Replicate sample preparation information (e.g., %CV or RPD, for duplicate preparation, of 10%–15%). - For nonstandard, nonvalidated studies: Discussion of at least three laboratory QC procedures and results in the methods section, supplementary materials, cited papers, or standardized laboratory protocol, including a combination of the procedures mentioned under “Good” and “Adequate” levels. |
| Deficient | 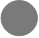 | Method  Same as Adequate, with the following exceptions:   - All papers must indicate the type of sample analyzed; for Deficient quality, may be liquid whole blood (collected in vacutainer tubes with ethylenediaminetetraacetic acid [EDTA] anticoagulant, 1 year storage in the refrigerator, or lithium heparin anticoagulant due to the lower stability of heparin) or a freeze-dried (2 years) blood sample.   NOTE: If samples have been stored for longer than noted above, recovery of storage stability spiked samples must be reported but results will not be evaluated. Additional information regarding storage stability samples is provided in “Useful Terms Defined”.   - Minimal methodological details provided in the paper, supplemental information, or cited papers; only one or two of the items from the aforementioned method detail list.   - For example, mentioning only the instrumental technique used for analysis, omitting all collection and storage procedures, analytical limits, or references for the sample preparation method.   NOTE: The analytical limits for nonstandard, nonvalidated methods must be provided for papers to be considered Good or Adequate, as it is necessary to determine whether a method was used with sensitivity levels appropriate to the matrix and that the method has been appropriately optimized by the analytical laboratory. If analytical limits for nonstandard, nonvalidated methods are not provided, the maximum rating possible is Deficient.  Quality Control  Studies will be evaluated as Deficient if they include the following:   - Use of NIST standard reference materials or commercial certified reference materials (CRMs) in non–blood-based matrices (e.g., NIST 2976, 3668, 1641e; several European Union Joint Research Centre [JRC] CRMs). - QC results fall outside of “Adequate” acceptance ranges but are not severe enough to warrant exclusion of the study (e.g., recovery of QC samples in the range 80%–85% or 115%–120%; variability of replicate samples 15%–20% RPD).   OR   - QC procedures and results not discussed in the paper or supplemental information.   NOTE: Where QC procedures are not described, requesting additional information from the corresponding author will be necessary because QC results are instrumental in gauging reliability of reported sample data. |
| Critically Deficient | 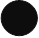 | Low proportion of samples (<50%) above the LOD OR LOD greater than median blood mercury concentration in the study population.  Method  If any of the following are true:   - Analysis of dried blood spots (DBS).   NOTE: To date, there have been virtually no studies that have explored the analysis of dried blood spot samples for mercury or methylmercury. Additionally, the field of dried blood spot analysis of metals is still in development, especially for quantitative applications.   - Use of inappropriate standard method (e.g., methods EPA 245.2 or 7471B).   NOTE: The above methods describe the analysis of bulk volumes of water for mercury content, so those and similar methods cannot be directly used for analysis of mercury in blood. It may be possible to adapt such methods for use with blood samples, but they should be treated as nonstandard, nonvalidated methods and detailed descriptions of the preparation and analysis methods must be provided.   - No collection, preparation, or analysis method details described in the paper, supplementary information, or cited reports.   NOTE: If no description of the preparation and analysis methods are provided, it is not possible to confirm that appropriate measures were taken to ensure the accuracy of results. Quality control results alone may not account for all essential method parameters in study samples (e.g., collection and storage measures).   - For ICP-MS or GC-ICP-MS studies: No internal standard reported.   NOTE: The use of internal standards is an essential aspect of ICP-MS analysis to appropriately account for instrument drift and matrix impact on analyte signals.  Quality Control   - QC results reveal concerns about reliability of measurements (e.g., recovery of QC samples outside 80%–120%, variability of replicate samples >20% RPD). - If after inquiry, it is found that no QC samples were analyzed.   NOTE: Analysis of quality control samples is essential to demonstrate the accuracy of chemical analyses and provide confidence in data quality and is generally considered a standard practice in analytical laboratories. QC samples can demonstrate that analytical accuracy is maintained even in complex sample matrices, such as blood. If no quality control samples were prepared by the same method and analyzed alongside study samples, then it is not possible to have confidence in the quality of analytical data generated in support of an epidemiology study. |

Supplemental Table 3. Evaluation of analytical methods for blood total and methylmercury in epidemiology studies

| Level | | Criteria |
| --- | --- | --- |
| Good | 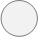 | Acceptable proportion of samples (>50%) above the limit of detection (LOD) OR LOD less than median hair mercury concentration in study population  Method  All papers must include a description of methodological factors:   - All papers must indicate the sample collection considerations including: age and sex of study participants, hair segment characteristics (e.g., length from scalp, length of hair analyzed). Studies should clearly indicate the area of the body from which hair was collected (e.g., scalp, underarm, pubis), which is important for interpreting results and comparing across studies. - Sample storage and shipment procedures must be described including sample containers/bags, tying procedures, etc. - Cleaning procedures for hair samples must be described. Acceptable washing procedures include deionized water, ionic or nonionic detergent solution, acetone, methanol, etc. Multiple washes or heat, or both, may be used below 65°C. An example standard cleaning technique is presented in IAEA Report 50 (IAEA/RL/50), “Activation analysis of hair as an indicator of contamination of man by environmental trace element pollutants.”a - For laboratories using a standard method: The paper will include the method number and a citation for a standard method for measuring mercury levels in hair (e.g., United States Department of Agriculture (USDA) Food Safety Inspection Service [FSIS] method MER for total mercury, Environmental Protection Agency (EPA) SW-846 Method 3200 for extraction only, EPA SW-846 method 6800 for Hg speciation only, European Union Consortium to Perform Human Biomonitoring on a European Scale [COPHES] D3.6 “Mercury: Determination in Scalp Hair” for total mercury only) OR a method description discussed as follows. - For laboratories using a nonstandard, validated method: The paper will provide a citation for a peer-reviewed report of the validation in the main body of the paper or in supplementary information (refer to “Useful Terms Defined” for more information on validation) OR a description of the method discussed, as follows. - For laboratories using nonstandard, nonvalidated methods: Nonstandard methods may perform acceptably if sufficient evidence of data quality (quality control [QC] results) is provided (as follows). For nonstandard methods, the report must provide a description of the method including the following:   - Analytical limits including LOD and/or the limit of quantitation (LOQ) must be reported for nonstandard, nonvalidated methods to obtain a Good rating.   - Combustion Atomic Absorption Spectroscopy (CAAS) refers to direct mercury analysis and may use several instruments, such as the Milestone Direct Mercury Analyzer (DMA) or the Nippon Mercury Analyzer (MA).     - For studies using CAAS for total mercury analysis, critical method variables that should be noted are time and temperature of sample drying step, charring/decomposition step of analysis, and amalgamator flash time and temperature.   - Sample stabilization measures (e.g., addition of gold or thiols during or after extraction; refer to ”Useful Terms Defined” for additional details).   - Sample preparation method including, but not limited to, the following:     - Hair sample drying method and conditions (e.g., temperature, time).     - Derivatization steps for gas chromatography-inductively coupled plasma-mass spectrometry (GC-ICP-MS) analysis or another speciation technique for MeHg.     - Oxidizing agents for cold vapor atomic fluorescence spectroscopy (CVAFS) and cold vapor atomic absorption spectroscopy (CVAAS) (e.g., potassium permanganate [KMnO_4_], nitric acid, or sulfuric acid for total mercury analysis).     - Reducing agent for CVAFS and CVAAS (e.g., tin chloride [SnCl_2_], sodium borohydride [NaBH_4_], sodium hypochlorite [NaClO_2_], hydroxylamine hydrochloride).     - For total mercury analysis only: Digestion reagents, temperatures (≤65°C for open vessel digestion or digestion without stabilizers mentioned previously), and times.     - Extraction solvent composition. - For speciation analysis (i.e., methylmercury): Separation steps before detection. Examples include the following:   - For liquid chromatography (LC; e.g., high-performance liquid chromatography [HPLC] or ultra-high-performance liquid chromatography [UHPLC]): Column type, eluent composition (e.g., mercaptoethanol, ethylenediaminetetraacetic acid [EDTA], cysteine), eluent gradient (or indicate whether an isocratic program [where the same eluent content was used through the run] was used), and injection volume.   - For gas chromatography: Transfer line temperature, column temperature program, and column identity.   - For distillation: Solvent and temperature. - Use of an appropriate internal standard (e.g., bismuth [Bi], praseodymium [Pr], holmium [Ho]; ICP-MS only; other organomercury compound [e.g., propylmercury, butylmercury; speciation by LC- or GC-ICP-MS only]):   - NOTE: Internal standard is not generally included in CVAAS/CVAFS/CAAS methods.   NOTE: the items listed above should be included, but due to the great variability in nonstandard methods, it is not necessary to evaluate the quality of all methodological factors, only ensure that they are included in the method description.  Quality Control  All papers (standard methods, validated methods, and nonstandard methods) must include a description of QC procedures and results performed to verify method performance and data quality for their study:   - At least three laboratory QC procedures and results in methods section, supplementary materials, cited papers, or standardized laboratory protocol including, but not limited, to the following:   - Hair-based certified reference material (CRM) (NIES CRM 13: Human Hair, IAEA-085 or -086: Human Hair (Methyl Mercury), Joint Research Centre CRM BCR-397: Trace elements in human hair for total mercury, Chinese CRM GBW 07601): % recovery information, 85–115%.   NOTE: As of this time, no NIST (National Institute of Standards and Technology) Standard Reference Materials are available for hair mercury.   - - Duplicate sample preparation and relative percent difference (RPD) results, <20% RPD.   - Representative blank sample analyses or chromatograms to demonstrate the absence of interferences.   - Recovery of spiked study samples, 85%–115%.   - Post-extraction spiked sample recovery or method blank spiked (MBS) samples, 85%–115%.   - Replicate QC precision (also called uncertainty, imprecision, repeatability, or reproducibility; or percent relative standard deviation [%RSD] or coefficient of variation [%CV], <20%, or correlation coefficient >0.85).   - Participation in an interlaboratory testing program with documented results for mercury in hair samples and “satisfactory” results.   NOTE: A wide range of interlaboratory testing programs is available for *trace* metals, but only ones that monitor mercury in hair, or that use standard methods, are relevant for assessing data quality.   - - Incurred sample reanalysis (ISR) to demonstrate reproducibility on different days, and RPD results, <20% RPD; refer to “Useful Terms Defined” for more information.   - Control charts (e.g., Bland-Altman, Levey-Jennings, Harrell-Davis, Shewhart) for QC samples of the same sample type, prepared by the cited method that show method performance over time (large sample populations only).   Note: If any of the above method parameters or quality control measures exhibit values that fall outside the range of values noted above, then the entire study should be categorized at the lowest quality level of the individual method parameter(s) or QC measure(s). |
| Adequate | 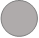 | Method  Same as Good, with the following exceptions:   - All papers may indicate the sample collection considerations including age and sex of study participants, hair segment characteristics (e.g., length from scalp, length of hair analyzed). Studies should clearly indicate the area of the body from which hair was collected (e.g. scalp, underarm, pubis), which is important for interpreting results and comparing across studies. - Sample storage and shipment procedures must be described including sample containers/bags, tying procedures, etc. - Cleaning procedures for hair samples must be described. Acceptable washing procedures include deionized water, ionic or nonionic detergent solution, acetone, methanol, etc. Multiple washes or heat, or both, may be used below 65°C. An example standard cleaning technique is presented in IAEA Report 50 (IAEA/RL/50), “Activation analysis of hair as an indicator of contamination of man by environmental trace element pollutants.”^a^ - For laboratories using standard methods: Samples analyzed by EPA SW-846 method 6800 (Hg speciation only) or method 7473 (total mercury only). - For nonstandard, nonvalidated methods: The report will comment on two or three methodological details above such as extraction times, cleaning times between sample analyses, instrumental technique, and so on. - Analytical limits including LOD and/or the limit of quantitation (LOQ) must be reported for nonstandard, nonvalidated methods to obtain an Acceptable rating. - For measurements of methylmercury: Determined methylmercury by subtracting inorganic mercury (iHg) from total mercury (THg). - For ICP-MS only: Use of less common or less appropriate internal standards such as terbium (Tb), rhodium (Rh), gallium (Ga), thallium (Tl), indium (In), yttrium (Y), or scandium (Sc).   Quality Control  Any Adequate analysis must include:   - For standard methods and validated methods (with literature citation): Discussion of one or two laboratory QC procedures and results in methods section, supplementary materials, cited paper, or standardized laboratory protocol including either:   - Reference material percentage recovery information (may be the certified reference materials [CRMs] mentioned earlier or another such as NIST Standard Reference Material [SRM] 3133 or 3177 for use in spiking total Hg only), recovery will fall in the range 80%–85% or 115%–120%.   - Replicate sample preparation information (e.g., %CV or RPD) for duplicate preparation of 20%–25%. - For nonstandard, nonvalidated studies: Discussion of at least three laboratory QC procedures and results in the methods section, supplementary materials, cited papers, or standardized laboratory protocol, including a combination of the procedures mentioned under “Good” and “Adequate” quality levels. |
| Deficient | 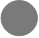 | Method  Same as Adequate except:   - For Deficient quality: All papers may indicate 1-2 sample collection considerations including age and sex of study participants, hair segment characteristics (e.g., length from scalp, length of hair analyzed). Studies should clearly indicate the area of the body from which hair was collected (e.g. scalp, underarm, pubis), which is important for interpreting results and comparing across studies. - Sample storage and shipment procedures must be described including sample containers/bags, tying procedures, etc. - Cleaning procedures for hair samples must be described. Acceptable washing procedures include deionized water, ionic or nonionic detergent solution, acetone, methanol, etc. Multiple washes or heat, or both, may be used below 65°C. An example standard cleaning technique is presented in IAEA Report 50 (IAEA/RL/50), “Activation analysis of hair as an indicator of contamination of man by environmental trace element pollutants.”^a^ - Minimal methodological details provided in the paper, supplemental information, or cited papers; only one or two of the items from the aforementioned method detail list.   - For example, only mentioning the instrumental technique used for analysis, omitting all collection, storage, and cleaning procedures, analytical limits or references for the sample preparation method.   NOTE: The analytical limits for nonstandard, nonvalidated methods must be provided for papers to be considered Good or Adequate, as it is necessary to determine whether a method was used with sensitivity levels appropriate to the matrix and that the method has been appropriately optimized by the analytical laboratory. If analytical limits for nonstandard, nonvalidated methods are not provided, the maximum rating possible is Deficient.  Quality Control  Studies will be evaluated as Deficient if they include the following:   - Use of NIST standard reference materials or commercial certified reference materials in non-hair-based tissue matrices (e.g., NIST 1575a [total mercury only], 1946, 1947, 2976, several European Union Joint Research Centre [JRC] CRMs). - QC results fall outside of “Adequate” acceptance ranges but are not severe enough to warrant exclusion of the study (e.g., recovery of QC samples in the range 75%–80% or 120%–125%; variability of replicate samples 25%–50% RPD).   OR   - QC Procedures and results not discussed in the paper or supplemental information.   NOTE: Where QC procedures are not described, requesting additional information from the corresponding author will be necessary because QC results are instrumental in gauging reliability of reported sample data. |
| Critically Deficient | 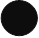 | Low proportion of samples (<50%) above the LOD OR LOD greater than median blood mercury concentration in the study population.  Method  If any of the below are true:   - Use of inappropriate standard method (e.g., methods EPA 245.1, EPA 245.2, EPA 245.7, EPA 7470A, EPA 1630, EPA 1631E).   NOTE: The above methods describe the analysis of bulk volumes of water for mercury content, so those and similar methods cannot be directly used for analysis of mercury in hair. It may be possible to adapt such methods for use with hair samples, but they should be treated as nonstandard, nonvalidated methods and detailed descriptions of the preparation and analysis methods must be provided.   - No collection, preparation, or analysis method details described in the paper, supplementary information, or cited reports.   NOTE: If no description of the preparation and analysis methods are provided, it is not possible to confirm that appropriate measures were taken to ensure the accuracy of results. Quality control results alone may not account for all essential method parameters in study samples (e.g., collection and storage measures).   - Cleaning, drying, or open-vessel extraction of samples at any temperature greater than 65°C except sample preparation for speciation analysis by distillation and closed‑vessel digestion of hair samples for total mercury analysis in the presence of sulfur-containing stabilizing agents.   NOTE: Mercury volatilizes at temperatures above 65°C in the absence of a stabilizer during sample preparation such as thiols or gold. Cleaning and drying procedures are not generally performed in the presence of such stabilizers and open vessel digestions will readily allow release of volatilized mercury.   - For ICP-MS or GC-ICP-MS studies: No internal standard reported.   NOTE: The use of internal standards is an essential aspect of ICP-MS analysis to appropriately account for instrument drift and matrix impact on analyte signals.  Quality Control   - QC results reveal concerns about reliability of measurements (e.g., recovery of QC samples outside 75%–120% or variability of replicate samples >20% RPD). - If after inquiry, it is found that no QC samples were analyzed.   NOTE: Analysis of quality control samples is essential to demonstrate the accuracy of chemical analyses and provide confidence in data quality and is generally considered a standard practice in analytical laboratories. QC samples can demonstrate that analytical accuracy is maintained even in complex sample matrices, such as blood. If no quality control samples were prepared by the same method and analyzed alongside study samples, then it is not possible to have confidence in the quality of analytical data generated in support of an epidemiology study. |

^a^Ryabukhin, Y. S. (1976). Activation analysis of hair as an indicator of contamination of man by environmental trace element pollutants (No. IAEA-RL--50). International Atomic Energy Agency.

Supplemental Table 4. Evaluation of analytical methods for hair total and methylmercury in epidemiology studies
